# Inhibition of FGFR3 upregulates MHC‐I and PD‐L1 via TLR3/NF‐kB pathway in muscle‐invasive bladder cancer

**DOI:** 10.1002/cam4.6172

**Published:** 2023-06-07

**Authors:** WenBo Wu, Lei Chen, GaoZhen Jia, QiLin Tang, BangMin Han, ShuJie Xia, Qi Jiang, HaiTao Liu

**Affiliations:** ^1^ Department of Urology Shanghai General Hospital Shanghai China; ^2^ Shanghai JiaoTong University School of Medicine Shanghai China

**Keywords:** bioinformatics, FGFR3, immune subtype, MIBC, NF‐KB pathway

## Abstract

**Background:**

Improving the potency of immune response is paramount among issues concerning immunotherapy of muscle‐invasive bladder cancer (MIBC).

**Methods:**

On the basis of immune subtypes, we investigated possible molecular mechanisms involved in tumor immune escape in MIBC. According to the 312 immune‐related genes, three MIBC immune subtypes were clustered.

**Results:**

Cluster 2 subtype is characterized by FGFR3 mutations and has a better clinical prognosis. However, the expression levels of MHC‐I and immune checkpoints genes were the lowest, indicating that this subtype is subject to immune escape and has a low response rate to immunotherapy. Bioinformatics analysis and immunofluorescence staining of clinical samples revealed that the FGFR3 is involved in the immune escape in MIBC. Besides, after FGFR3 knockout with siRNA in RT112 and UMUC14 cells, the TLR3/NF‐kB pathway was significantly activated and was accompanied by upregulation of MHC‐I and PD‐L1 gene expression. Furthermore, the use of TLR3 agonists poly(I:C) can further improve the effect.

**Conclusion:**

Together, our results suggest that FGFR3 might involve in immunosuppression by inhibition of NF‐kB pathway in BC. Considering that TLR3 agonists are currently approved for clinical treatment as immunoadjuvants, our study might provide more insights for improving the efficacy of immunotherapy in MIBC.

## 1 INTRODUCTION

Among all malignancies of the urinary tract, bladder cancer (BC) is the most common. According to the American Joint Committee on Cancer, BC can be categorized into non‐muscle‐invasive bladder cancer (NMIBC; PT_a_‐PT_1_) or muscle‐invasive bladder cancer (MIBC; ≥PT_2_), and approximately 30% of newly diagnosed bladder cancers are MIBC.^1^ Patients with MIBC require radical cystectomy or systemic chemotherapy, which significantly reduces their quality of life. In addition, tumor progression and distant metastasis may occur in 50% of MIBC patients.^2^ Therefore, improving the efficiency of comprehensive treatment of MIBC is a challenging problem. Recently, immune checkpoint blockade (ICB) therapy has shown shown long‐term tolerance and safety in the treatment of malignant tumors. However, there are about 70%–80% of patients may not respond to immune checkpoint inhibitors, and the responsible mechanism is unclear.^3^ Studies have shown that immune subtypes can be identified in gastric cancer, colon cancer, glioblastoma, and pancreatic adenocarcinoma, showing significant differences in the immunotherapy response.^4^, ^5^, ^6^, ^7^, ^8^ Although there have been some studies on the classification of BC, there are few reports on how to improve the efficacy of MIBC immunotherapy.^9^, ^10^, ^11^ In this study, we identified MIBC immune subtypes and further studied the molecular mechanism of immunosuppression, which will help provide guidance for the immunotherapy of BC.

The fibroblast growth factor receptor 3 (FGFR3) protein, encoded by a gene on chromosome 4, is a tyrosine kinase that plays an important role in cell growth. FGFR3 gene mutation is most common in uroepithelial carcinoma (18%), followed by uterine carcinosarcoma (14%), esophageal cancer (5%), ovarian cancer (5%), and endometrial cancer (4%).^12^, ^13^ FGFR3 mutations are primarily associated with lower tumor grades and stages in uroepithelial bladder cancer, and patients with FGFR3 mutations generally have a better clinical prognosis.^14^, ^15^, ^16^ In addition, studies have shown differences in response rates of immune checkpoint blockade (ICB) between patients with FGFR3 mutation and those with wild‐type FGFR3.^18^ However, the detailed molecular mechanism by which FGFR3 affects the immune microenvironment in BC remains unclear. An important reason for ICB resistance is that tumor cells cannot activate T cells due to insufficient expression of antigen‐presenting molecules.^19^ Antigen‐presenting molecules include mainly MHC‐I and MHC‐II; MHC‐I can present intracellular polypeptide antigens on the cell surface for recognition by CD8^+^ T cytotoxic lymphocytes, which is a key step for activating the role of CD8^+^ lymphocytes.^20^ Studies have also shown that a lack of antigen‐presenting molecules can lead to dysfunction of CD8^+^ T cells during ICB therapy.^21^ In addition, low expression levels of MHC‐I molecules can be observed in 40%–90% of human tumors and are closely associated with poor prognosis.^23^, ^24^ A study on the correlation between the expression levels of MHC‐I molecules and the number of infiltrating lymphocytes in tumor tissues have confirmed that downregulation of MHC‐I molecules is involved in immune escape.^22^ Therefore, enhancing MHC‐I levels is a promising strategy to improve ICB efficacy, especially for cancers with low MHC‐I levels. The expression of MHC‐I molecules is strictly regulated in tumors, and the enhancer A region in their transcription factor binding sites can be recognized by NFkB.^25^, ^26^ The classical NF‐kB pathway can be activated by a variety of receptors, such as toll‐like receptors (TLRs), the tumor necrosis factor receptors (TNFR), and cytokine receptors and their ligands (such as IL‐1 and LPS), and participates in immune response regulation.^27^, ^28^ In this study, we found that the expression of TLR3 was significantly increased by silencing with siRNA‐FGFR3, accompanied by activation of the NF‐kB pathway and increased expression of MHC‐I and PD‐L1 molecules. Considering that TLR3 agonists are currently approved for clinical treatment as immunoadjuvants, our study might provide more insights for improving the efficacy of immunotherapy in MIBC.

## 2 MATERIALS AND METHODS

### 2.1 Data processing

BC patient datasets containing data for a total of 412 patients with BC and 19 samples of normal tissue adjacent to carcinoma were downloaded from the TCGA platform (https://cancergenome.nih.gov/). The data included clinical prognosis information, mRNA‐seq data (count format), and single‐nucleotide variation (SNV) data for different patients. The mRNA‐seq data were uniformly subjected to log2 (*x* + 0.01) transformation to facilitate comparative analysis between groups. Next, immune‐related genes were downloaded from IMMport platform (https://www.immport.org/resources). The GSE84732 and GSE125547 datasets were downloaded from GEO (www.ncbi.nlm.nih.gov/geo/). All data sets were extracted and analyzed using R X64 4.1.1 software.

### 2.2 Identification of MIBC immune subtypes

The edgeR package^29^ was used to preprocess the data. MIBC samples were selected according to T stage, and 371 MIBC samples and 19 paracancerous samples were selected. The differentially expressed genes (DEGs) were defined with log2FC (fold change) > 1.2 and *p* < 0.05 as the screening criteria. The DEGs were intersected with 1509 immune‐related genes to obtain the immune‐related DEGs in MIBC. The Consensus Cluster Plus R package was used for clustering according to the expression characteristics of the immune‐related DEGs; the K‐means clustering algorithm was selected for clustering, and the maximum cluster number (maxK) was set to 9. Optimal cluster sizes were determined using the cumulative distribution function (CDF). With log2FC > 1.5 and *p* < 0.05 as the screening criteria, the top 30 genes in each subtype were selected as the marker genes and analyzed with the pheatmap R package.

### 2.3 Clinical prognosis of different subtypes of MIBC

The survival^30^ package was used for survival analysis. The differences in overall survival (OS), disease‐specific survival (DSS), disease‐free survival (DFS), and progression‐free survival (PFS) among the 3 subtypes were compared.

### 2.4 Characteristics of the immune microenvironment in different subtypes of MIBC

The CIBERSORT R script was used to analyze the distribution of immune cells in the different subtypes of MIBC, and the R packages (corplot, vioplot, and ggplot2) were used to visualize the results. The ESTIMATE score, immune score, and stromal score were analyzed with the ESTIMATE R package and visualized with the ggplot2 package. Comparisons between the target genes were shown on boxplots via the ggpubr package.

### 2.5 Mutation characteristics in different subtypes of MIBC

The maftools^31^ R package was used to analyze gene mutations in patients with the different subtypes, and the waterfall plots showed the top 30 genes with the highest mutation frequency.

### 2.6 KEGG and GO enrichment analyses of the DEGs

Metascape^32^ (http://metascape.org.) was used for KEGG and GO enrichment analyses.

### 2.7 Patients

All the procedures were approved by the Ethics Committee of the Shanghai General Hospital, and patients in this study provided the informed consent. The mutations of FGFR3 gene were verified by gene‐panel (ChosenOne‐599, CHOSENONE).

### 2.8 GSEA and single‐cell RNA sequencing analysis

The CAMOIP platform^33^ (https://www.camoip.net/) can be used to perform Gene set enrichment analysis (GSEA) based on TCGA cohort and the Mariathasan cohort. Antigen presentation, the inflammatory response, and NF‐kB pathways were analyzed in BC patients with and without FGFR3 mutation. The TIGER platform^34^ (http://tiger.canceromics.org/) was used for single‐cell RNA sequencing analysis (Dataset ID:BTCC1, Dataset Name: Chen, Z., Nat Commun 2020 Oct 08).

### 2.9 Cell culture and FGFR3 siRNA transfection in vitro

The RT112 cell line used in this study was purchased from the Cell Bank of the Chinese Academy of Sciences (Shanghai, China). The culture medium RPMI 1640 contained 10% fetal bovine serum and 1% penicillin (Gibco). These cells were cultured in an incubator containing 5% CO_2_ at 37°C. For transfection of siRNA, the following siFGFR3‐1 sequences were used^35^: sense (5′‐3′) CCGUAGCCGUGAAGAUGC, antisense (5′‐3′) AGCAUCUUCACGGCU ACGG. The siFGFR3‐2 sequences: sense (5′‐3′) CCUGC GUCGUGGAGAACA, antisense (5′‐3′) UUGUUCUCCACGACGCAGG. siFGFR3‐1 and siFGFR3‐2 could knock down both wild‐type and FGFR3‐TACC3 expression in RT112 cells. The cells were seeded into a six‐well plate at a density of 5 × 10^5^ cells/well. When the cell confluence reached 60–70%, siFGFR3‐1 and siFGFR3‐2 were transfected into the cells using a Lipo3000 kit (Invitrogen). After siRNA transfection, the cells were cultured for 48 h. Polyinosinic:polycytidylic acid [poly(I:C)] and IKK‐inhibitor (HY‐138537) were purchased from MCE (MedChem‐ Express).

### 2.10 Western blot analysis

The protein content was quantified by BCA kit. Protein samples (5 μL) were loaded onto a 15% SDS–PAGE gel and then transferred onto a PVDF membrane. The 5% skim milk was used to block the membrane (room temperature RT, 90 min), and then, the membrane incubated with a primary antibody (anti‐P65, 1:1000, No. 8242; anti‐TLR3, 1:3000, No. 6961, CST; anti‐HLA‐A/B/C, 1:1000,No. 15240‐1‐A; anti‐PD‐L1, 1:2000, No. 66248‐1‐Ig; anti‐FGFR3, 1:1000, No. 66954‐1‐lg; anti‐GAPDH, 1:30000, No. 60004‐1‐lg, Proteintech) at 4°C overnight and incubated with an enzyme‐conjugated secondary antibody (1:2000, HRP‐labeled Goat Anti‐Rabbit A0208; Anti‐Mouse IgG A0216, Beyotime) at RT for 90 min. GAPDH or Histone H3 proteins were used as the loading control for normalization, and the enhanced chemiluminescence (ECL) reagent (BeyoECL, Beyotime) was used for development.

### 2.11 Immunofluorescence and IHC assay

The RT112 cells were plated and fixed on slides. Then, cells were permeabilized with 0.02% Triton at RT for 10 min. Then, the slides were incubated with a primary antibody against human P65 (CST, Cat No. 8242) overnight (12 h) at 4°C. For immunofluorescence of tissues, sections were dewaxed by xylene and then dehydrated in ethanol. After blocked with goat serum, the specimens were incubated with primary antibodies against human FGFR3 (Proteintech, 66,954‐1), HLA‐A/B/C (Proteintech, 15,240‐1), CD8 (Proteintech, 66,868‐1) at 4°C overnight. Then, Goat Anti‐Rabbit IgG H&L conjugated secondary antibodies were applied, and DAPI was used for nuclear staining. At last, the incubated slides were visualized with an FV1000‐IX81 fluorescence microscope (Olympus). The IHC of PD‐L1 was performed by VENTANA PD‐L1(SP263)Assay.

### 2.12 Statistical analysis

The GraphPad Prism 6.0 software was used for statistical analysis. Student's *t*‐test was used for comparison between groups and *p* < 0.05 indicated statistical significance.

## 3 RESULTS

### 3.1 Identification of MIBC subtypes based on immune‐related genes

**FIGURE 1:**
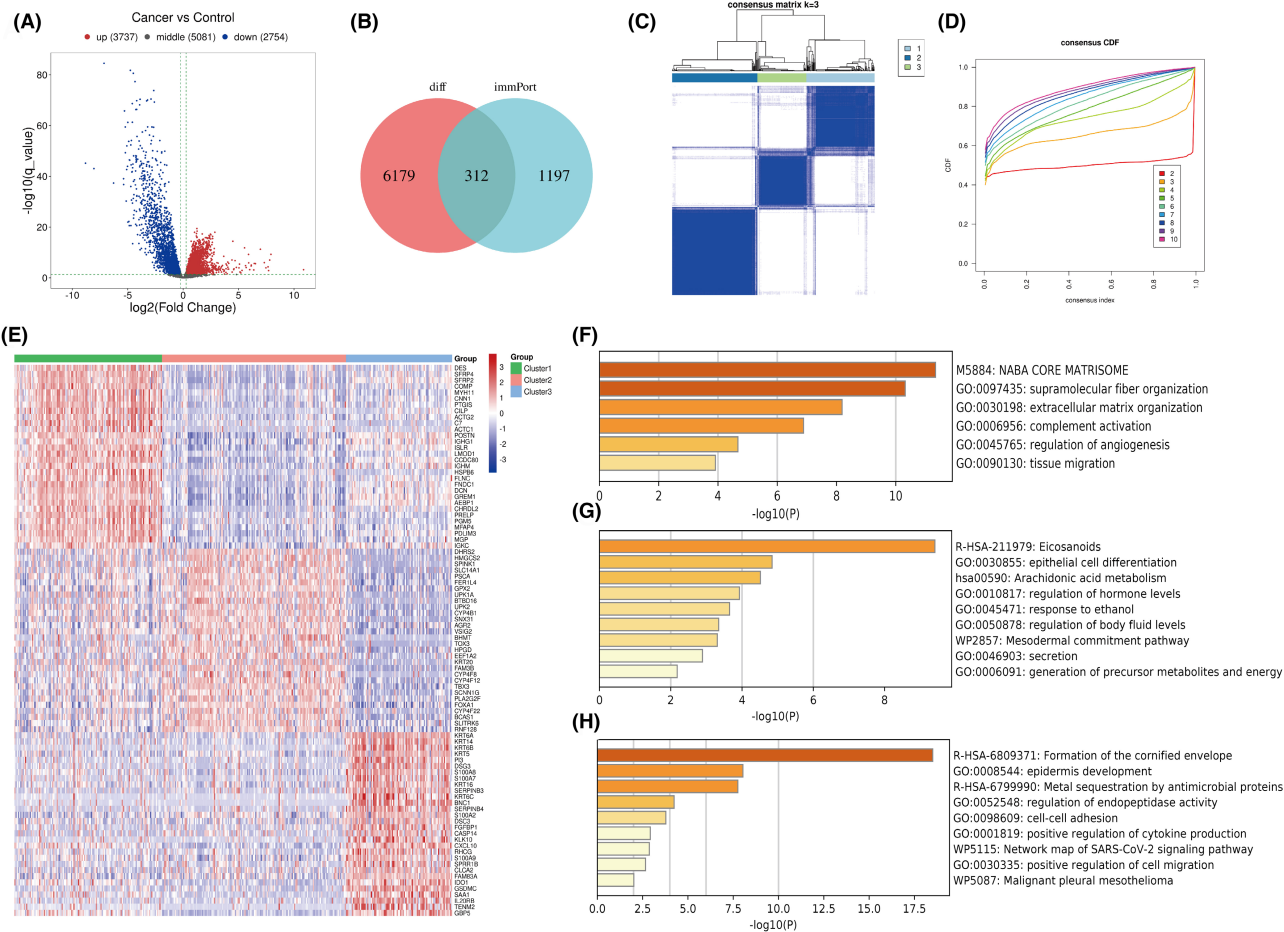
Identification of MIBC subtypes based on immune‐related Genes. (A) Volcano plot of differentially expressed mRNAs in MIBC. (B) Venn diagram demonstrated the intersection set of DEGs and immune‐related genes. (C, D) Consensus matrix heatmap and CDF curve. (E) Heat map of 30 marker genes in each of the three subtypes. (F–H) Enrichment analysis of 30 marker genes.

DEGs were identified from samples of TCGA‐MIBC, as shown in Figure 1A. There were 6491 DEGs in MIBC tissues compared with normal tissues: 3737 upregulated genes and 2754 downregulated genes. A total of 312 immune‐related differentially expressed genes were identified by intersecting the differentially expressed genes with the immune‐related genes (Figure 1B). Based on these 312 immune‐related genes, the immune subtypes were identified via consensus cluster analysis. Based on the cluster graph, the discrimination was best when *k* = 3, in which the white and blue modules were the purest (Figure 1C). In addition, *k* = 3 was optimal when considering the consensus score of the CDF curve (Figure 1D). Based on the clustering results, the three subtypes were identified as Clusters 1/2/3. Enrichment analysis of 30 marker genes in each of the three subtypes was conducted to clarify the main characteristics of each subtype (Figure 1E–H). The results showed that the Cluster 2 subtype was characterized by epithelial differentiation (GO:0030855: Epithelial cell differentiation). Cluster 3 was characterized by epidermal and keratin differentiation (R‐HSA‐6809371: Formation of the cornified envelope, GO:0008544: Epidermis development). The enrichment of extracellular matrix components was higher in the Cluster 1 subtype (GO:0030198: Extracellular matrix organization; GO:0045765: Regulation of angiogenesis).

### 3.2 Immune characteristics of immune subtypes

**FIGURE 2:**
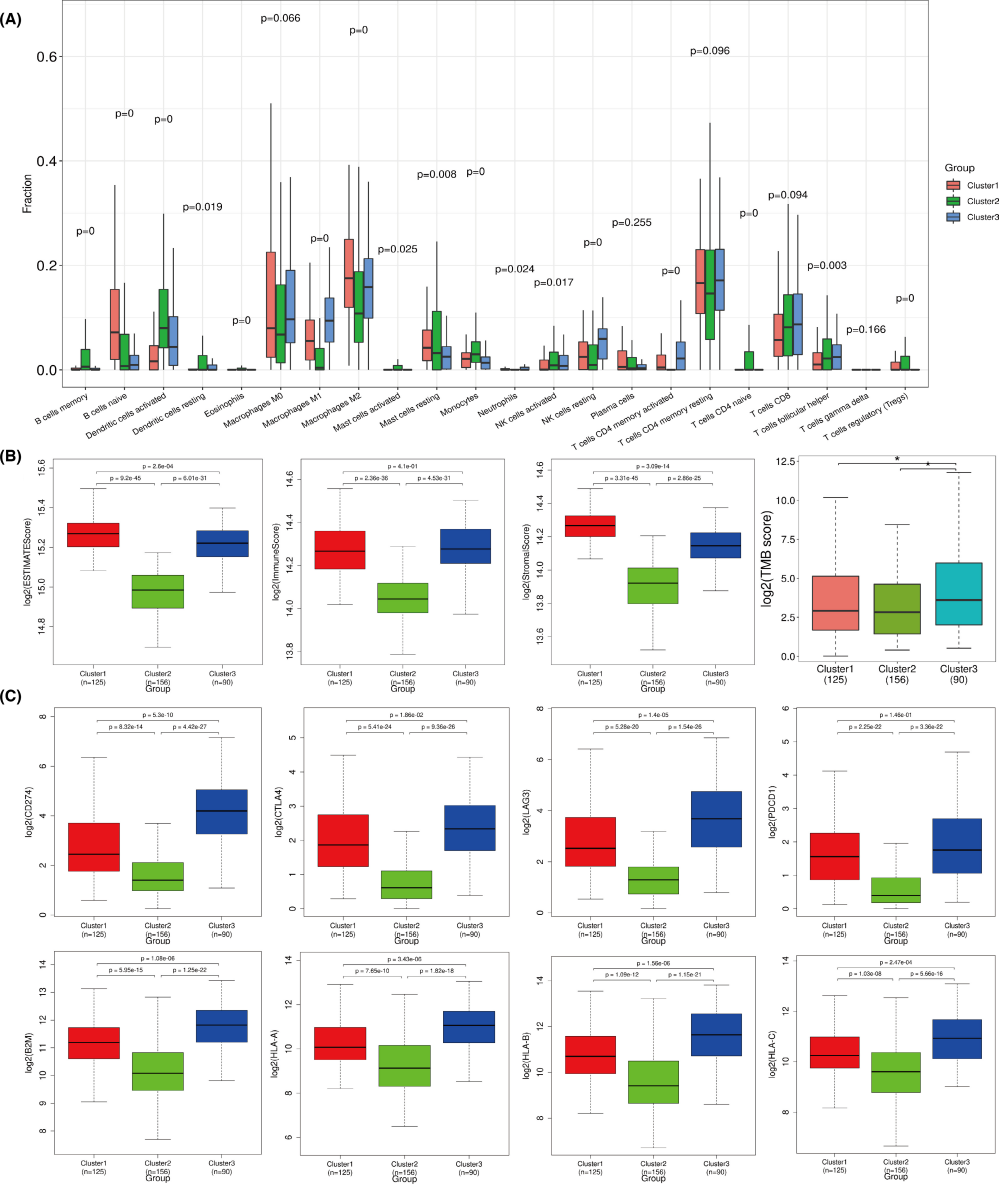
Immune characteristics of the three immune subtypes. (A) The infiltration of 22 kinds of immune cells in the three subtypes of MIBC. (B) Microenvironment score by ESTIMATE. (C) Expression of immune checkpoint genes and antigen presentation genes in the three subtypes of MIBC.

The characteristics of immune infiltration cells in the three subtypes of MIBC was studied via CIBERSORT. The results (Figure 2A) showed a significantly higher number of the memory B cells, activated dendritic cells, monocytes, naive CD4 positive T cells, and regulatory T cells (Tregs) in the Cluster 2 than in Cluster 1 or Cluster 3 (*p* < 0.01). On the contrary, macrophages (M_0_/_1_/_2_) had the lowest distribution in the Cluster 2 subtype (*p* < 0.01), but the M_2_/M_1_ ratio was highest in this subtype. In summary, the number of Tregs and the M_2_/M_1_ ratio suggested that the microenvironment of the Cluster 2 subtype was immunosuppressed.^36^, ^37^ In addition, Cluster 3 contained the highest distributions of CD4^+^ activated memory‐T cells and M_1_ macrophages (*p* < 0.01). The distributions of naive B cells and M_2_ macrophages were the highest in Cluster 1 (*p* < 0.01). Moreover, we used the ESTIMATE algorithm^38^ to assess the tumor microenvironment and found that Cluster 2 had the lowest ESTIMATE score, immune score, and stromal score (*p* < 0.01). As compared to other clusters, Cluster 1 showed significantly higher ESTIMATE and stromal scores (*p* < 0.01), suggesting that the proportion of stroma was the largest in the microenvironment of Cluster 1 subtype. Cluster 3 had the highest immune score but showed no significant difference from Cluster 1 (Figure 2B). In immunotherapy, tumor mutation burden (TMB), which determines the total number of mutations in tumor tissue, has been considered a biomarker.^39^, ^40^ In this study, the TMB score in Cluster 3 was the highest (*p* < 0.01), while the TMB score in Cluster 2 was the lowest (Figure 2B). In addition, we examined the expression of PDCD1, PD‐L1 (CD274), CTLA4, LAG3, and PD1 (PDCD1) in different subtypes. The results showed that Cluster 2 had the lowest expression levels of all immune checkpoint genes, and the same results were observed for the antigen presentation genes (HLA‐A/B/C and B2M; *p* < 0.01). In conclusion, the Cluster 2 subtype may conform to the characteristics of an “immune desert” and has a strong immune escape ability.

Clinical prognosis and gene mutation characteristics of the three immune subtypes.

**FIGURE 3:**
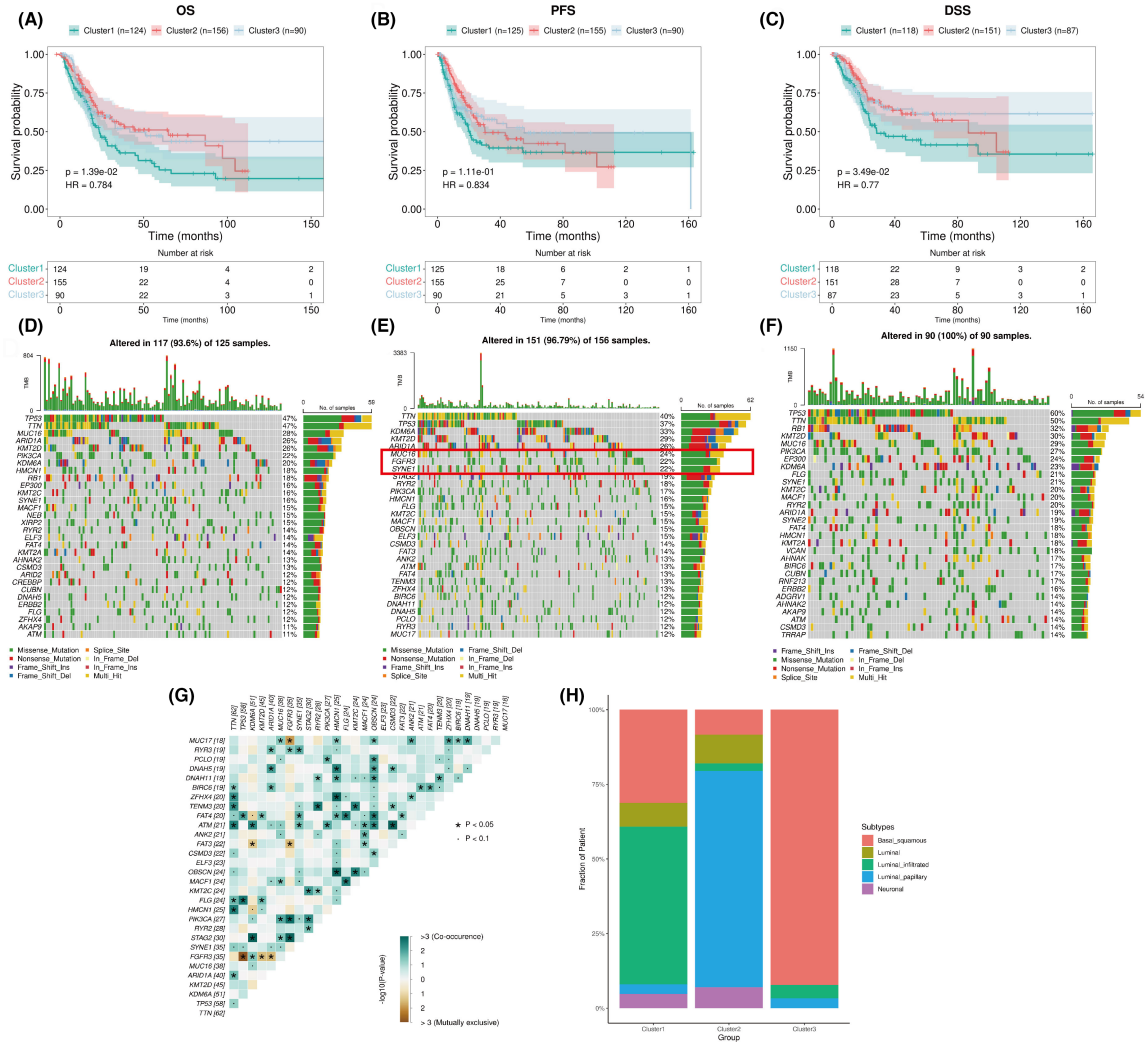
Clinical prognosis and gene mutation characteristics of the three immune subtypes. (A–C) Clinical prognosis (OSl, DSS, PFS). (D–F) Top 30 mutated genes in the three subtypes. (G) Correlation analysis of mutated genes. (H) Comparison with TCGA subtypes.

The results showed that there were significant differences in OS and DSS among the three subtypes. Patients in Cluster 2 and 3 subtypes had better OS and DSS when compared patients in Cluster 1 subtype. However, PFS showed no significant difference among the three subtypes (Figure 3A–C). Figure 3D/E/F shows the top 30 mutated genes in the three subtypes, among which P53 and TNN had the highest mutation frequency in the three groups. However, the mutation frequency of FGFR3 in Cluster 2 was significantly higher than that in the other groups. Furthermore, mutations in FGFR3 were mutually exclusive with mutations in TP53, KMT2D, ARID1A, MUC17, and FAT3 but co‐occurred with mutations in KDM6A, RYR3, PIK3CA, and STAG2 (Figure 3G). We also compared the three immune subtypes with the five classical subtypes in TCGA, and the results showed that Cluster 2 was mainly composed of the luminal‐papillary subtype, which is also characterized by FGFR3 mutations.^41^ Cluster 3 was mainly composed of the basal‐squamous subtype, and Cluster 1 was mainly composed of the luminal‐infiltrated subtype and the basal‐squamous subtype (Figure 3H). Based on the mutation of FGFR3, we divided clinical data of MIBC patients from TCGA into FGFR3‐mut and FGFR3‐wt groups (Table S1). A significant correlation was found between FGFR3‐mut and earlier pTNM stages (*p* = 0.011).

### 3.3 Validation in clinical samples with FGFR3 mutation

**FIGURE 4:**
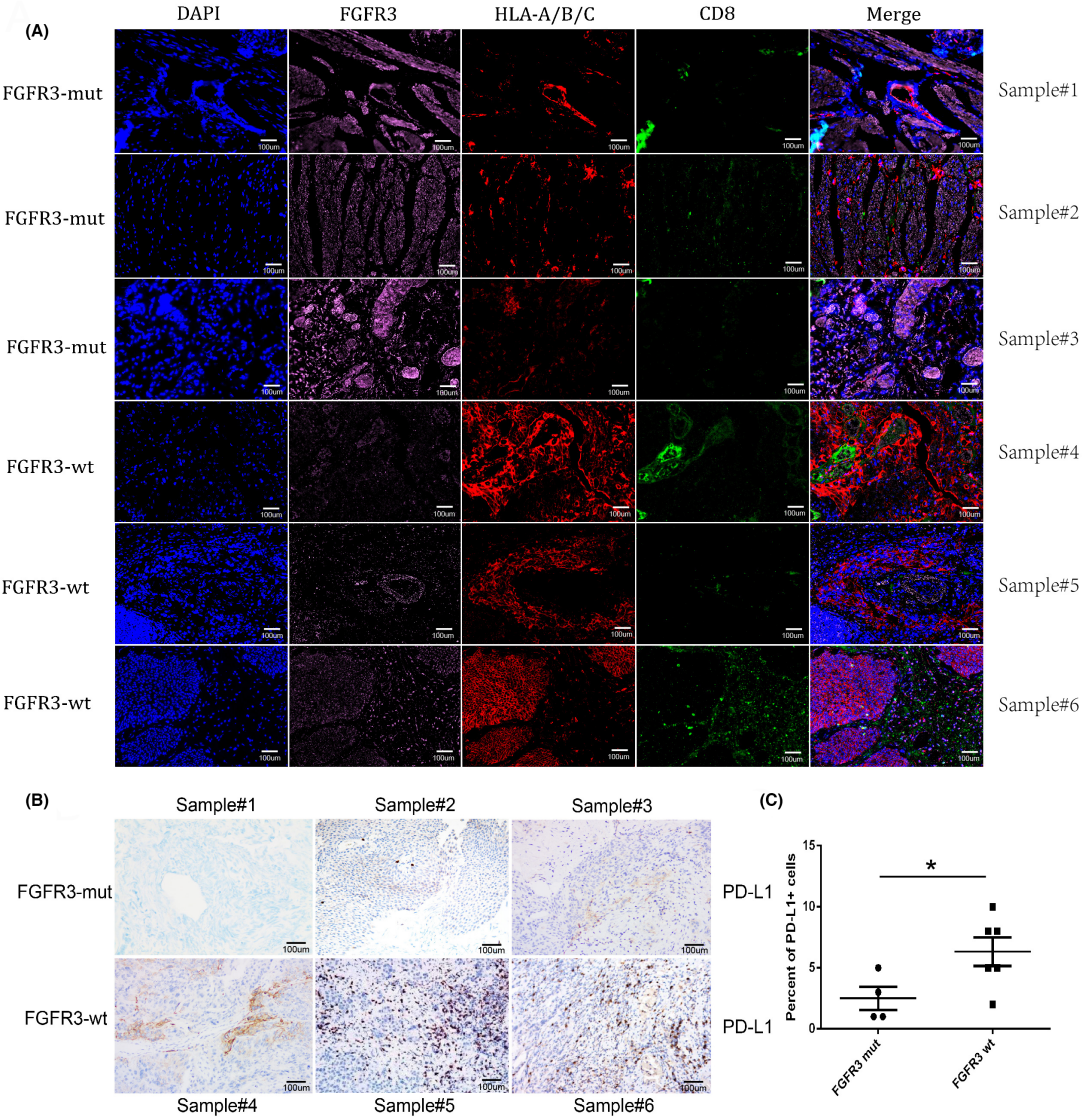
Validation in clinical samples with FGFR3 mutation. (A) Immunofluorescence staining of DAPI (blue) FGFR3(pink), HLA‐A/B/C (red), and CD8(green). (B) Immunohistochemistry of PD‐L1. (C) FGFR3‐wt showed a higher proportion of PD‐L1 positive cells (*p < 0.05).

Then, we sought to validate our findings in clinical patient samples. Immunofluorescence staining of FGFR3, HLA‐A/B/C, and CD8 were detected in MIBC tissues with mutated FGFR3 or non‐mutated FGFR3, separately. Strong pink fluorescence indicates higher expression level of FGFR3 in mutated group, indicating that the samples with activating FGFR3 alterations expressed higher levels of FGFR3. At the same time, the red fluorescence intensity was weaker in the mutated group than in the non‐mutated group, suggesting that expression of FGFR3 is inversely correlated with HLA‐A/B/C. Besides, CD8 was used as a marker to reflect the distribution of CD8^+^ T cells and showed no significant difference between the two groups (Figure 4A). Finally, immunohistochemistry of PD‐L1 was also performed, and the results showed that the number of PD‐L1 positive cells was higher in the non‐mutated group (Figure 4B/C).

### 3.4 FGFR3 knockdown activates the NF‐kB pathway

**FIGURE 5:**
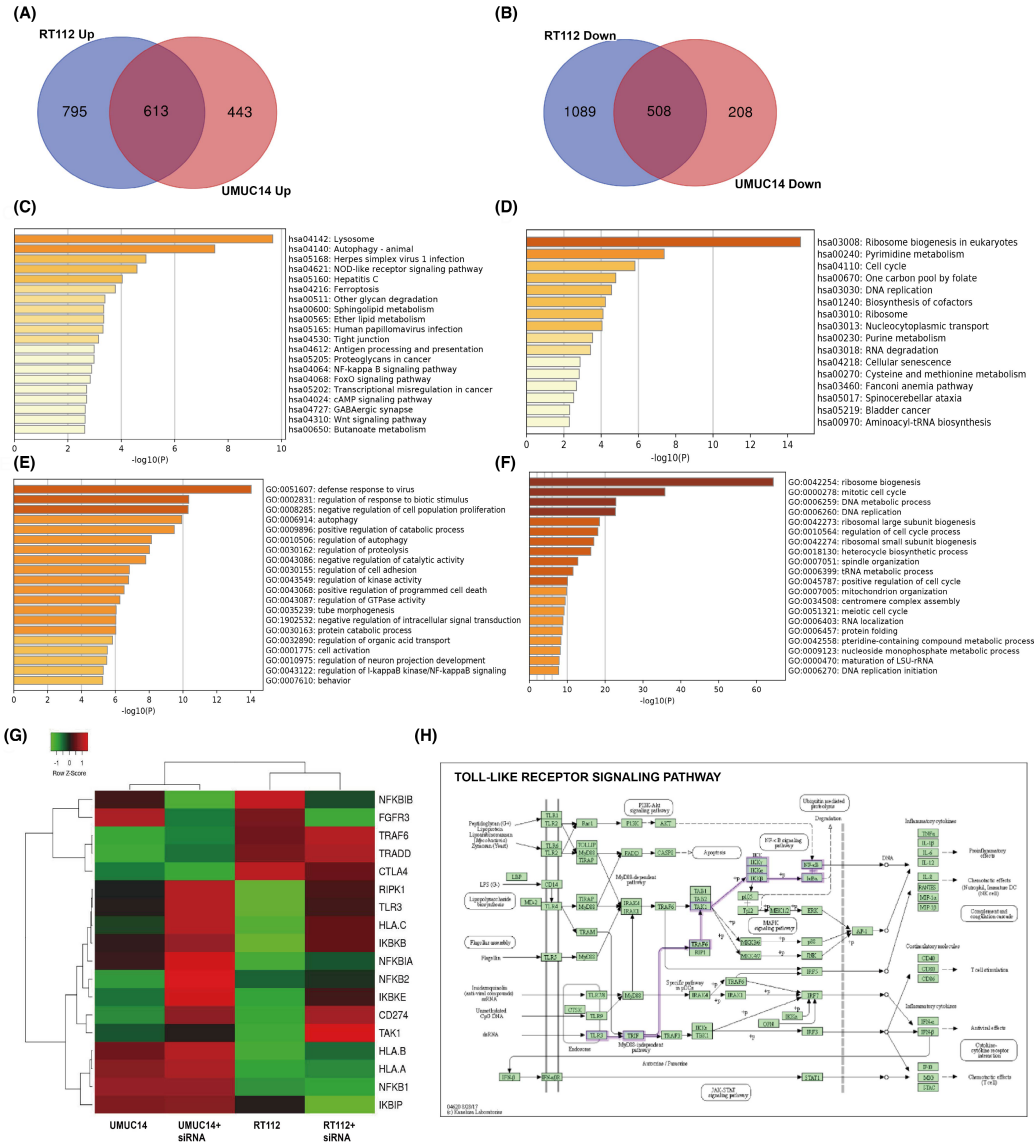
FGFR3 knockdown activates the NF‐kB pathway. (A, B) DEGs after FGFR3 knockout by siRNA‐ FGFR3 were identified in the RT112 and UMUC‐14 bladder cancer cell lines. (C, E) KEGG and GO analysis of upregulated genes. (D, F) KEGG and GO analysis of downregulated genes. (G) Heat map of genes in NF‐kB signaling pathway. (H) KEGG pathway analysis indicated that the TLR3‐NF‐kB pathway was activated.

To further investigate the mechanisms of immunosuppression by FGFR3, the GSE84732 and GSE125547 datasets were analyzed. Differentially expressed genes after FGFR3 knockout by siRNA‐FGFR3 were identified in the RT112 and UMUC‐14 BC cell lines with FGFR3 mutations. The results showed that FGFR3 knockout led to an increase in 613 genes and a decrease in 508 genes in both cell lines (Figure 5A,B). KEGG enrichment analysis showed that immune‐related pathways such as the antigen processing and presentation pathway and the NF‐kappa B signaling pathway were activated. Moreover, cell apoptosis‐related pathways such as the autophagy and ferroptosis pathways were also significantly activated after FGFR3 knockout (Figure 5C). In contrast, the cell cycle and DNA replication pathways were inhibited (Figure 5D). In addition, the GO analysis results also showed the activation of the NF‐kB pathway (GO:0043122: regulation of I‐KappaB kinase/NF‐kappaB signaling; Figure 5E). The heatmap (Figure 5F) shows that the mRNA levels of NFKB2, IKBKB IKBKE, RIPK1, TRAF6, TAK1, and TRADD were significantly increased and that the antigen presentation genes HLA‐A/B/C and immune checkpoint gene PD‐L1 (CD274) were also upregulated. In our study, Toll‐like receptor (TLR3) was significantly upregulated after FGFR3 knockout, and was significantly upregulated after FGFR3 knockout, and KEGG pathway analysis indicated that the TLR3‐NF‐kB pathway was activated (Figure 5F). In addition, studies have shown that MHC‐I (HLA/B/C) molecules are downstream effectors of the TLR3‐NF‐kB pathway.^42^, ^43^ In conclusion, we speculated that interference with FGFR3 expression could activate the NF‐kB pathway by TLR3 and enhancing the expression of antigen presentation genes.

### 3.5 Single‐cell RNA sequencing analysis of FGFR3 in BC

**FIGURE 6:**
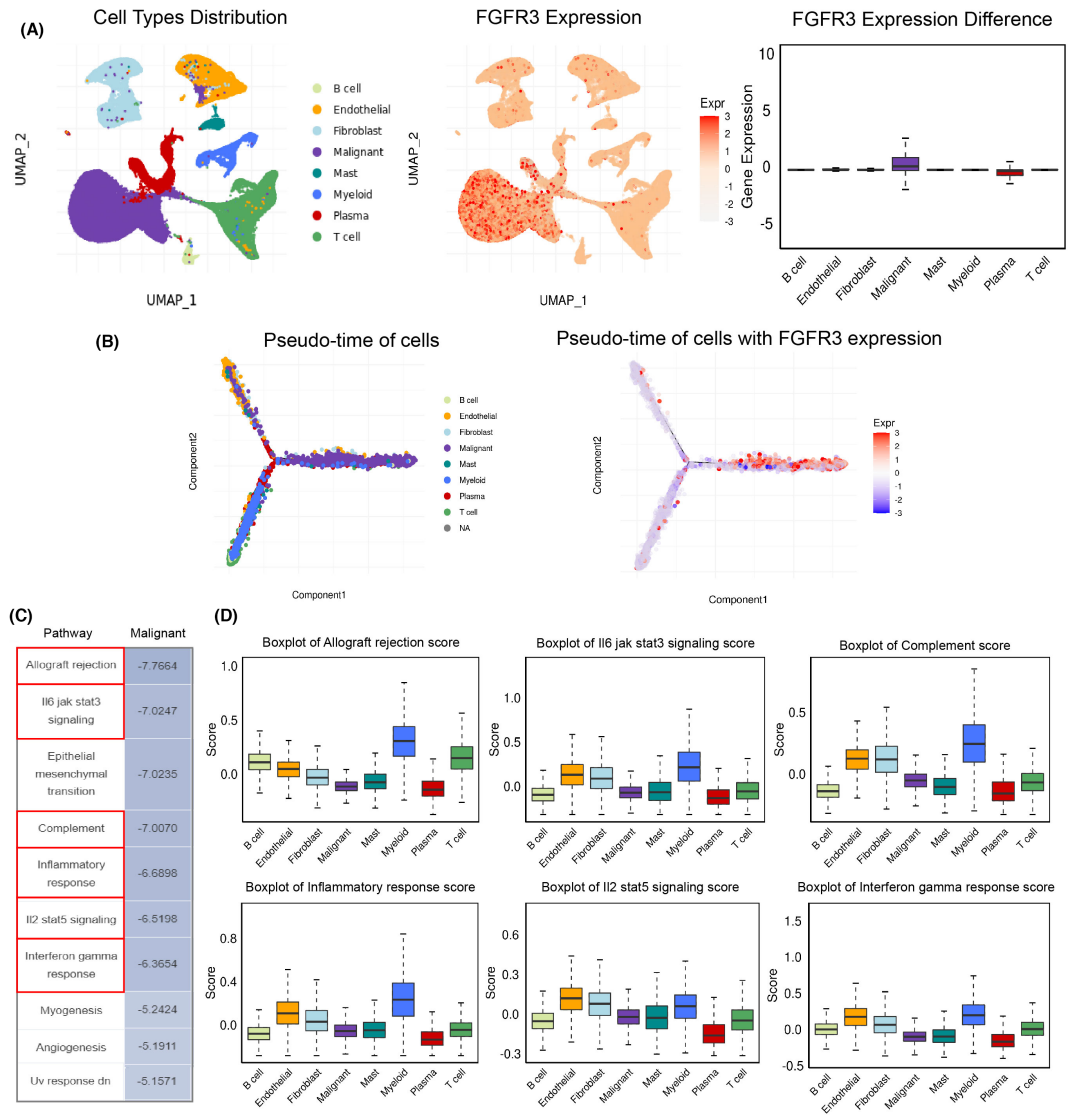
Single‐cell RNA sequencing analysis of FGFR3 in BC. (A) Cell types distribution and FGFR3 expression in different cell types. (B) Single‐cell trajectory analysis. (C) The 10 most negatively correlated pathways in malignant cells. (D) The enrichment of immune or inflammation‐related pathways in different cell types is shown as box‐plot diagrams.

Overexpression or activating mutation of FGFR3 is the most frequent genetic alteration in luminal‐papillary BC. A total of 8 BC samples(2 low‐grade and 6 high‐grade) were included in the single‐cell analysis. Results showed that FGFR3 expression was mainly detected in tumor malignant cells (Figure 6A). Meanwhile, the single‐cell trajectory analysis showed that the FGFR3 expression mainly occurs in mature and differentiated malignant cells (Figure 6B). Furthermore, we examined the correlation between FGFR3 expression and tumor hallmark pathways in malignant cells. The 10 most negatively correlated pathways were showed in Figure 6C, and six of these were immune/inflammation‐related pathways (Allograft rejection, IL6 jak stat3 signaling, Complement, Inflammatory response, IL2 stat5 signaling, Interferon gamma response; Figure 6D). Among these, the most significant negatively correlated pathway is allograft rejection, which is mainly involved in activation of innate immunity and antigen presentation of MHC I and MHC II.

### 3.6 GSEA in patients with BC

**FIGURE 7:**
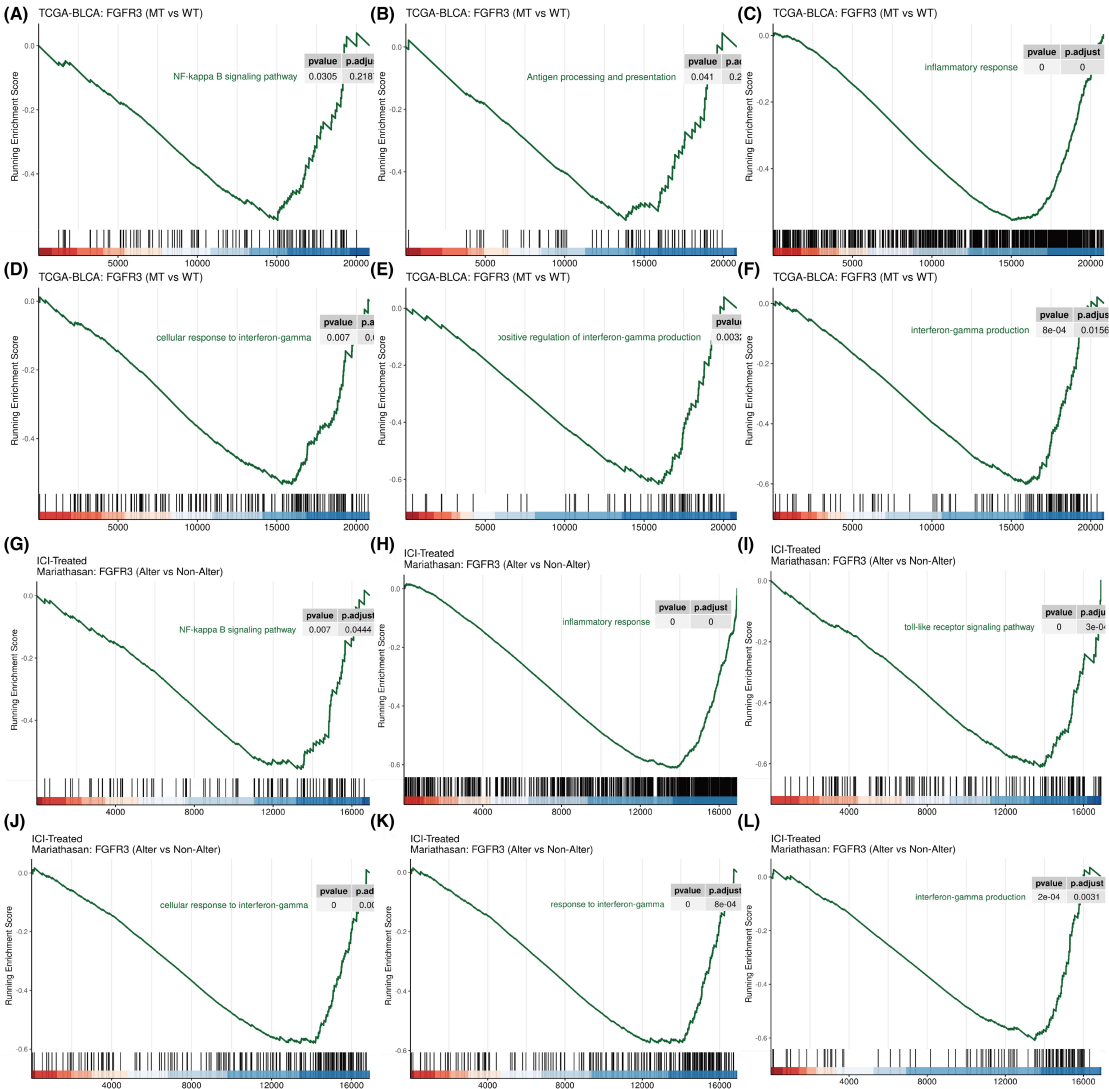
GSEA in the TCGA (A‐F) and Mariathasan (G‐L) cohorts.

To gain further insight into correlation between FGFR3 mutation and immune‐related pathways, GSEA was performed in patients with FGFR3 mutation and wild‐type FGFR3 in the TCGA and Mariathasan cohorts. A significant difference was found between patients with FGFR3 mutations and patients without FGFR3 mutations in terms of NF‐kappaB Signaling Pathway, Antigen Processing and Presentation, Inflammatory response and Interferon‐gamma pathways (Figure 7A–F). Similar results were found in the Mariathasan cohort, which contains patients who received ICB therapy. The NF‐kappaB signaling pathway, inflammatory response, interferon gamma‐related pathway, and TLRs signaling pathway were weakly enriched in patients with FGFR3 mutation (Figure 7G–L).

In conclusion, GSEA showed that FGFR3 mutation was negatively correlated with the NF‐kB pathway, antigen presentation pathway, and Toll‐like receptor pathway, suggesting that FGFR3 may inhibit these pathways. In addition to the GSEA within these two cohorts, the correlation between FGFR3 and related genes was also analyzed. Based on the results, FGFR3 mRNA expression levels were significantly and negatively correlated HLA‐A/B/C, NFKB2, and CD274(PD‐L1) mRNA levels (Figure S1).

### 3.7 FGFR3 knockout upregulated the HLA‐A/B/C and PD‐L1 via TLR3/NF‐kB signaling pathway

**FIGURE 8:**
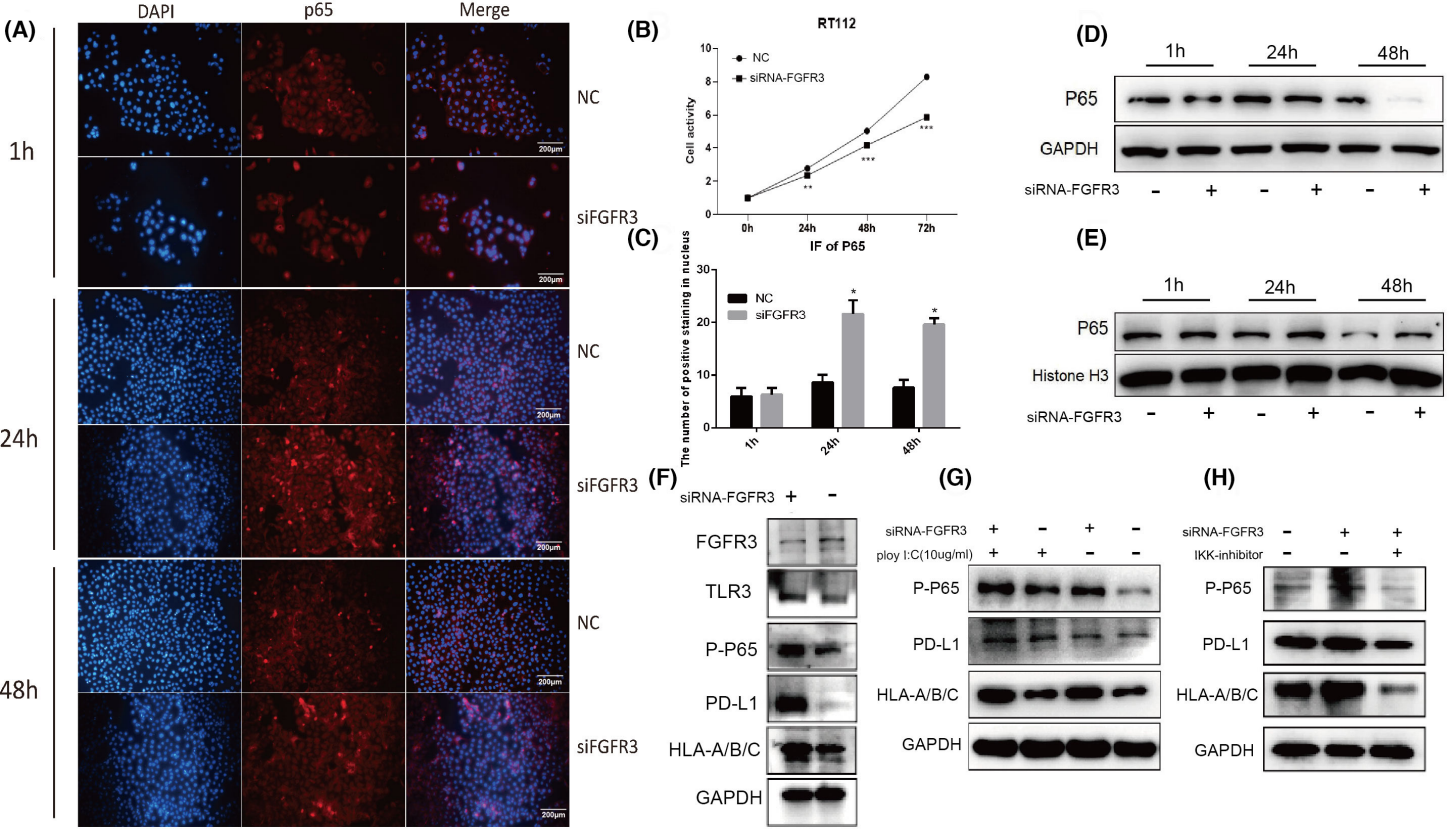
FGFR3 knockout activated the NF‐kB signaling pathway through TLR3 in vitro. (A/C) Immunofluorescence of P65 protein. (B) CKK8 assay in RT112 cells after FGFR3 knockdown. (D, E) Western blot assay of P65 protein in cytoplasm and nucleus. (F) WB showed that the FGFR3 protein level was decreased after knockout, while the protein levels of TLR3, P‐P65, PD‐L1, and HLA‐A/B/C were increased. (G) Poly(I:C) could further activate NF‐kB pathway and enhance the expression levels of the downstream proteins HLA‐A/B/C and PD‐L1. (H) IKK‐inhibitor could block NF‐kB activation and decrease the downstream proteins HLA‐A/B/C and PD‐L1.

RT112 cells with knockdown of FGFR3 showed dramatic reductions in proliferation in CKK8 assays. (Figure 8B). Then, we analyzed the NF‐kB pathway in the RT112 cell line after FGFR3 knockout via western blot analysis and an immunofluorescence assay. Entry of the P65 protein into the nucleus indicates activation of the NF‐kB pathway. The immunofluorescence results showed that the level of P65 was increased in the nucleus at 24 and 48 h after FGFR3 knockout (Figure 8A/C). The levels of the P65 protein in the nucleus and cytoplasm were determined by WB analysis (Figure 8D,E). The results also showed that the nuclear/cytoplasmic ratio of the P65 protein was increased at 24 and 48 h, suggesting that the NF‐kB pathway was activated. Following knockout, the protein level of FGFR3 was decreased, while the levels of TLR3, P‐P65, PD‐L1, and HLA‐A/B/C were increased (Figure 8F). The synthetic dsRNA analogue poly(I:C) is the strongest stimulator of TLR3, and we found that the poly(I:C) could further activate NF‐kB pathway and enhance the expression of the downstream proteins HLA‐A/B/C and PD‐L1. Conversely, IKK‐inhibitor could block NF‐kB activation and decrease the downstream proteins HLA‐A/B/C and PD‐L1 (Figure 8G,H). These results indicated that HLA‐A/B/C and PD‐L1 were downstream proteins in the NF‐kB pathway, and the TLR3‐NFkB‐HLA(A/B/C)/PD‐L1 pathway was activated after FGFR3 knockdown in RT112 cell line.

## 4 DISCUSSION

Currently, with the development of genomic analysis approaches, the study of tumor subtypes has become a research focus. The classical classification system for MIBC is the TCGA classification. In this study, 412 MIBC patients were reclassified into 5 subtypes based on different genetic characteristics: luminal‐papillary, luminal, luminal‐infiltrated, neuronal, and basal‐squamous.^40^ In addition, according to the immune microenvironment, three major immune phenotypes have been proposed in most solid tumors: “immune inflamed,” “immune excluded,” and “immune desert.”^34^ In our study, we selected MIBC patients in TCGA and identified three immune subtypes through unsupervised consensus clustering based on the expression characteristics of 312 immune‐related differentially expressed genes. Then, we analyzed the immune microenvironment and marker genes of the three immune subtypes of MIBC and found that Cluster 1 had the highest stromal score despite the infiltration of immune cells, reflecting the characteristics of the “immune excluded” immunophenotype. The lowest distribution of infiltrated immune cells was found in Cluster 2, conforming to the characteristics of the “immune desert” immunophenotype. Finally, Cluster 3 simply exhibited high infiltration of immune cells, showing the characteristics of the “immune inflamed” immunophenotype. These results indicate that dividing MIBC into three immune subtypes is plausible and our classification based on immune‐related DEGs is feasible. In addition, the expression of antigen presentation genes (HLA‐A/B/C) and immune checkpoint genes (CD274, CTLA4, LAG3 and PDCD1) in the Cluster 2 subtype was lowest, indicating the characteristics of “immune escape.” MHC‐I and MHC‐II molecules play an antigen‐presenting role in the immune response, in which MHC‐I molecules can recognize CD8+ T lymphocytes and are crucial in the antitumor process.^20^ Besides, tumor immune escape associated with loss of MHC‐I occurs frequently in solid tumors, according to growing evidence.^22^ We therefore speculate that there is a severe “immune escape” in Cluster 2‐subtype MIBC. Further analysis of gene mutations revealed that there were unique gene mutations in the Cluster 2 subtype, among which the mutation frequency of FGFR3 was the highest. The fibroblast growth factor receptor (FGFR) is a widely distributed transmembrane tyrosine kinase receptor and has four subtypes (FGFR1‐4), among which FGFR3 is the most commonly mutated in BC.^12^ FGFR3 alterations (mutations or translocations) occur in approximately 70% of NMIBCs and 20% of MIBCs, with S249C mutation and FGFR3‐TACC3 fusion being the most common. In FGFR3 mutant MIBC samples, immunofluorescence and immunohistochemistry showed lower expression of MHC‐I and PD‐L1, indicating a strong negative correlation. In vitro, the RT112 bladder cancer cell line was derived from a human bladder tumor and expresses the FGFR3‐TACC3 fusion protein, while the UMUC‐14 cell line expresses an endogenous activated mutant form of FGFR3 (FGFR3‐S249C).^33^, ^44^ According to our analysis, 613 genes appeared co‐upregulated in these two cell lines after FGFR3 knockout, while 508 genes appeared co‐downregulated. Then, KEGG and GO enrichment analyses showed that immune‐related pathways, such as the NF‐kB pathway, antigen presentation pathway, and Toll‐like receptor pathway, were significantly activated after FGFR3 knockout, suggesting that FGFR3 mutation may be a crucial cause of “immune escape” in MIBC. Studies have shown that FGFR3 inhibits the innate immune system by inhibiting interferon and TNF‐ɑ expression and affects adaptive immunity by inhibiting lymphocyte infiltration.^16^, ^17^ Although clinical studies have shown that FGFR3 mutations are associated with the immunotherapy response in BC, the underlying mechanism remains unknown.^18^, ^45^, ^46^ In addition, in a study of zebrafish, the authors found that overexpression of FGFR3 attenuated the activity of the kinase TBK1 in the NF‐kB pathway, thereby inhibiting IFN expression and immune responses.^47^ In our study, FGFR3 knockdown significantly increased the expression of TLR3, a member of a classical pathway for NF‐kB pathway activation. Besides, MHC‐I genes and immune checkpoint genes were also significantly upregulated. The NF‐kB pathway is an important pathway in immune and inflammatory responses and is closely involved in the release of interferon and the expression of antigen presentation genes.^25^, ^26^, ^41^, ^48^, ^49^ TLR3 usually functions as a tumor suppressor gene, and studies in prostate cancer and breast cancer showed that TLR3 can inhibit apoptosis, and TLR3 positive patients have a lower risk of metastasis.^50^, ^51^ The TLR3‐agonist poly(I:C) has been developed to simulate pathogen infection and promote immune system activation to enhance anticancer therapy and has shown significant therapeutic effects in preclinical models of melanoma, kidney cancer, and colorectal cancer.^52^, ^53^, ^54^ In our study, a combination of poly(I:C) and FGFR3 intervention further activated the NF‐kB pathway and promoted MHC‐I gene expression in vitro, indicating a new strategy to improve the immune response in MIBC patients with FGFR3 mutation. It is worth noting that erdafitinib (JNJ‐ 42756493), extremely potent competitive inhibitors of FGFR, have recently been approved by Food and Drug Administration (FDA) for treatment in MIBC patients with FGFR3 mutation. Whether FGFR3 inhibitors combined with TLR3 agonists can better improve the immune response and the efficacy of combination therapy with ICB drugs require further validation.

In conclusion, by identifying different immune subtypes of MIBC and analyzing the underlying molecular mechanism, we found that FGFR3 may be the crucial cause of immune escape in MIBC. Furthermore, FGFR3 intervention can significantly activate the TLR3/NF‐kB pathway to promote the expression of MHC‐I and PD‐L1 molecules. Considering that TLR3 agonists are promising in clinical treatment as immunoadjuvants, our results may provide guidance for developing novel immunotherapy strategies for MIBC patients with FGFR3 mutations and promoting individualized tumor immunotherapy in the future.

## AUTHOR CONTRIBUTIONS

**Lei Chen:** Writing – original draft (equal). **GaoZhen Jia:** Formal analysis (equal); methodology (equal). **QiLin Tang:** Methodology (equal); software (equal). **BangMin Han:** Investigation (equal); project administration (equal). **ShuJie Xia:** Investigation (equal); project administration (equal). **HaiTao Liu:** Funding acquisition (equal); supervision (equal). **Qi Jiang:** Funding acquisition (equal); investigation (equal); supervision (equal). **WenBo Wu:** Project administration (equal); writing – original draft (equal).

## FUNDING INFORMATION

This study was supported by the National Natural Science Foundation of China (No. 81972371 and No. 81972407).

## CONFLICT OF INTEREST STATEMENT

The authors declare no competing financial interests.

## CONSENT FOR PUBLICATION

Not applicable.

## Supporting information


Figure S1

Table S1

## Data Availability

The datasets presented in this study can be found in online repositories (http://www.cancer.gov/tcga; https://www.ncbi.nlm.nih.gov/geo/). The names of the repositories and accession number(s) can be found in the article. All the data obtained and analyzed during our research can be provided by the corresponding author upon reasonable request.
