# Supplementary material for: Inhibition of FGFR3 upregulates MHC‐I and PD‐L1 via TLR3/NF‐kB pathway in muscle‐invasive bladder cancer
Source: Cancer Med. 2023 Jun 7;12(14):15676–90. doi: 10.1002/cam4.6172 (PMC10417096; doi:10.1002/cam4.6172)
Supplement: Supplementary file 1 — Figure S1 [file CAM4-12-15676-s002.docx]

**Supplementary materials:**

Based on the FGFR3 median expression, samples in TCGA or Mariathasan cohort were divided into two groups.The gene expression levels of TLR3, HLA-A/B/C, NFKB1/2, NFKBIA/B, CD274 and PDCD1were compared between the two groups.


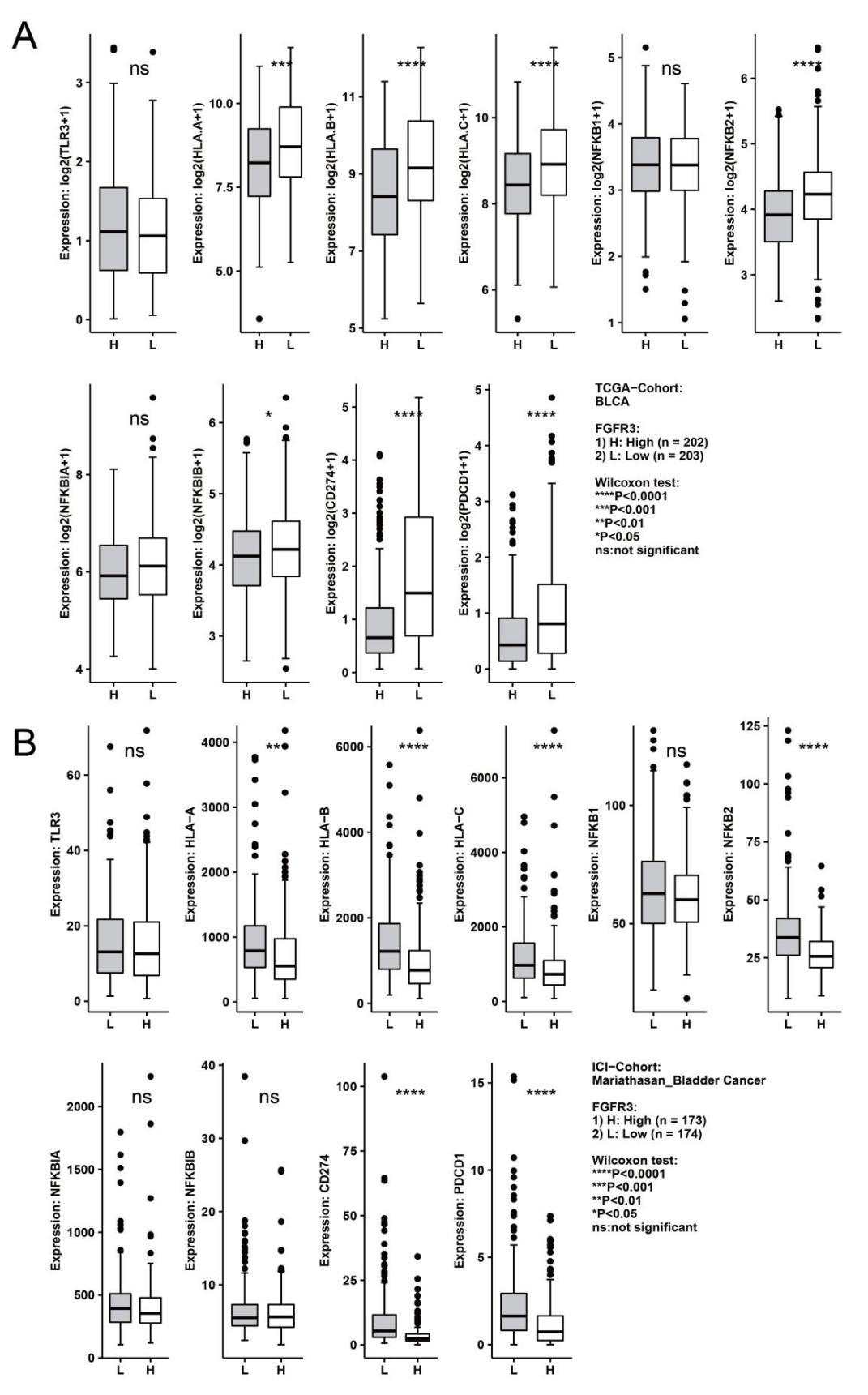


FIGURE S1 Expression levels of immuno-related genes in the two groups were compared. (A)TCGA-Cohort BLCA.(B)ICI-Cohort Mariathasan_Bladder Cancer.
